# Videographic analysis of flight behaviours of host-seeking *Anopheles arabiensis* towards BG-Malaria trap

**DOI:** 10.1371/journal.pone.0220563

**Published:** 2019-07-31

**Authors:** Elis P. A. Batista, Salum A. Mapua, Halfan Ngowo, Nancy S. Matowo, Elizangela F. Melo, Kelly S. Paixão, Alvaro E. Eiras, Fredros O. Okumu

**Affiliations:** 1 Laboratory of Innovation Technologies in Vector Control, Department of Parasitology, Biological Sciences Institute, Federal University of Minas Gerais, Belo Horizonte, Brazil; 2 Environmental Health and Ecological Sciences Department, Ifakara Health Institute, Ifakara, Tanzania; 3 School of Life Sciences, University of Keele, Staffordshire, United Kingdom; 4 School of Environment and Life Sciences, University of Salford, Manchester, United Kingdom; 5 Institute of Biodiversity, Animal Health and Comparative Medicine, University of Glasgow, Glasgow, United Kingdom; 6 University of Basel, Basel, Switzerland; 7 Swiss Tropical and Public Health Institute, Basel, Switzerland; 8 School of Public Health, Faculty of Health Sciences, University of the Witwatersrand, Parktown, Republic of South Africa; University of Crete, GREECE

## Abstract

The BG-Malaria trap (BGM) is an adaptation of the well-known BG-Sentinel trap (BGS) with greater trapping efficiencies for anopheline and culicine mosquitoes. Its continued optimization requires greater understanding of mosquito flight behaviors near it. We used three high-resolution infrared cameras (68 frames/second) to track flight behaviors of laboratory-reared *Anopheles arabiensis* females in vicinity of the BGM in comparison with BGS. Additional comparisons were done for BGM at 20, 40 and 80cm heights, and for BGMs baited with Ifakara blend plus CO_2_, CO_2_ alone, or no bait. More mosquitoes were observed near BGM than BGS. Both BGMs installed 20cm above the floor and baited with CO_2_ received more visits by host-seeking mosquitoes than the other BGMs evaluated in their respective experiments. Trap designs, height and attractants all influence mosquito activity in vicinity of the traps which can be readily visualized using infrared cameras to accelerate trap development and testing. The greater activity of host-seeking mosquitoes near BGM than BGS supports the proven superiority of BGM traps in field and semi-field settings.

## Introduction

As the world confronts renewed challenges from mosquito-borne diseases such as Zika virus, yellow fever, malaria, filariasis and Chikungunya virus, mosquito surveillance is becoming increasingly important both at country and regional levels. Effective trapping systems are particularly important to support initiatives such as malaria elimination, which now require reliable surveillance systems as core-interventions [1]. So far, there is no method more effective for collecting anophelines than the human landing catch (HLC), which is not only cumbersome, expensive and difficult to standardize, but also exposes the volunteer collectors to potentially infectious mosquito-borne pathogens. Recent advances such as the electric grid traps [2, 3], human-baited double net traps [4] and the MosqTent [5] address many of the HLC-related challenges but still require actual human collectors. Besides, the results can be affected by inherent differences in attractiveness of volunteers. To address this challenge, synthetic attractants mimicking human odors [6] can be used.

Examples of odour-baited traps previously used for malaria vectors include the Suna trap [7], the Ifakara odour-baited station [8], the MMX trap [9, 10], the mosquito landing box [11], the BG-sentinel (BGS) [12] and the BG-Malaria (BGM) [13]. The BGM trap is a promising adaptation of the BGS and has been demonstrated to effectively sampling malaria vectors *Anopheles darlingi* in Brazil [13, 14] and *Anopheles arabiensis* and *Anopheles funestus* in Tanzania [15, 16]. Since initial conception [13], the BGM has been improved by adding new mosquito retention systems [14] and using new synthetic attractants [15, 16]. Further optimization of the BGM, however, requires greater understanding of mosquito flight behaviors near it, e.g., how they approach, how long they spend in the vicinity and how they enter the trap.

Knowledge of how mosquitoes approach and enter a trap allows the improvement of capture mechanisms. Video tracking systems provide a wide range of possibilities to examine mosquito flight behaviors around traps and therefore help in improve trapping efficacies. For example, analyses of mosquito flight tracks showed different capture efficiencies among different traps [17] and change in trap orientation resulted in different flight patterns, followed by contrasting short-range attractiveness [18]. Knowing the mosquito flight dynamics in vicinities of traps can thus be exploited to achieve significant improvements in tools for vector surveillance. Here, we used infrared cameras and a video-tracking software to asses flight behaviours of laboratory-reared *Anopheles arabiensis* females in the vicinity of BGM and BGS traps. Data is used to elucidate how the mosquitoes approach the trap so as to improve trapping efficiencies of BGM.

## Methods

### Mosquitoes

Laboratory-reared *An*. *arabiensis* female mosquitoes were obtained from a colony maintained at Ifakara Health Institute since 2009. The mosquitoes larvae were fed with Tetramin fish food and reared under standard insectary conditions (29±1°C, 80±5% RH and 12:12h photoperiod). In a separate room with average temperatures of ~27°C and relative humidity of 70–90%, adult mosquitoes were kept in 30 x 30 x 30cm cages and fed with 10% sucrose solution daily. To propagate the colony, the adult female mosquitoes were fed also on bovine blood by a polytetrafluoroethylene-based membrane artificial feeding method [19], every two days. Mosquitoes used in the tests were those not previously blood-fed, were 3–8 days old, and had access to sugar only until 6h before experiments.

### BG-Malaria and BG Sentinel traps

**Fig 1:**
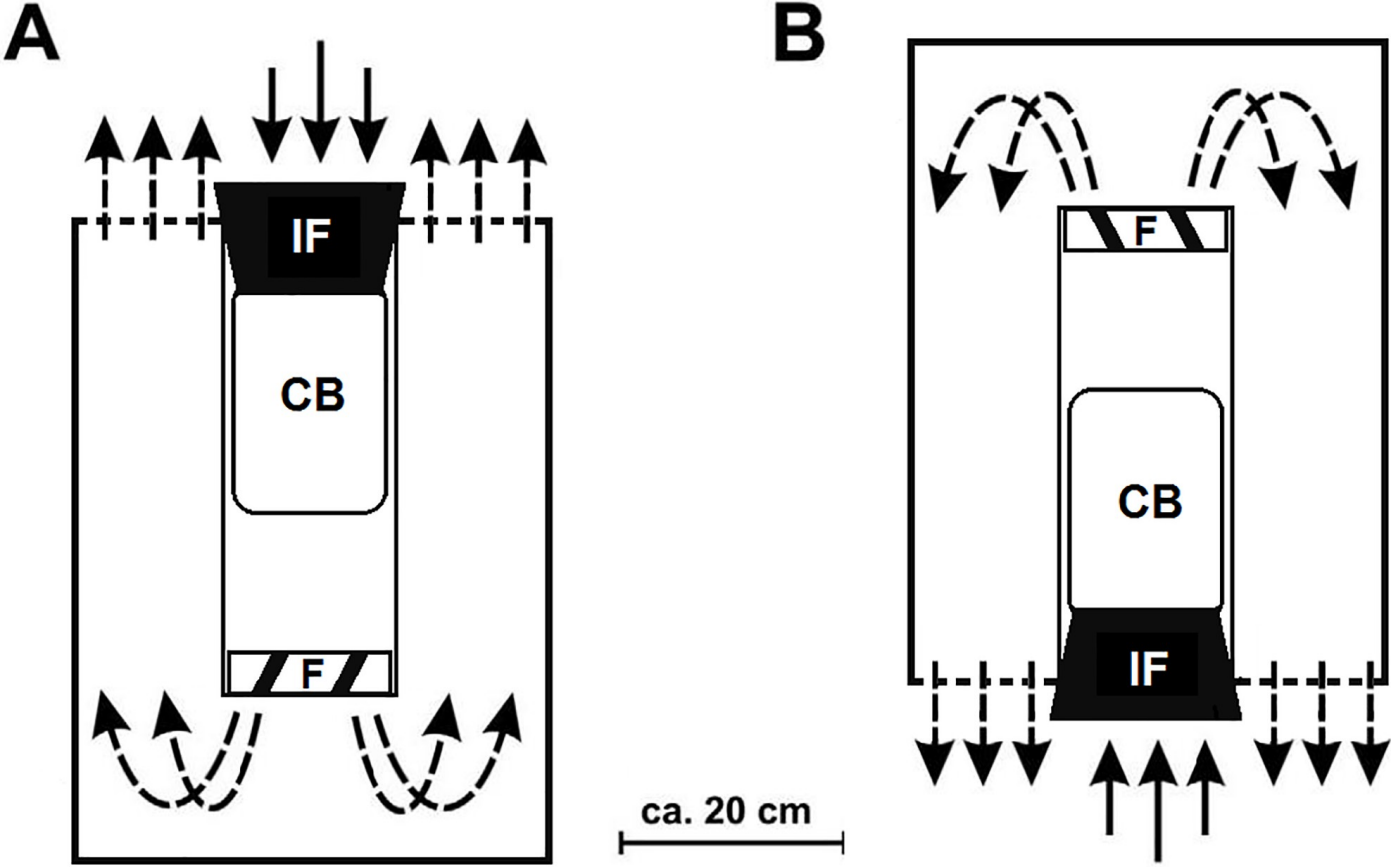
Illustration of the airflow direction (arrows) of the BG-Sentinel (A) and BG-Malaria (B) traps. IF = Intake funnel; CB = Catch Bag; F = Fan. Adapted from Batista et al., [16].

The BG-Malaria trap (BGM) is an adaptation of the widely-used BG-Sentinel trap (BGS) [13, 16]. Both traps are cylindrical and measure 35cm in diameter and 40cm in height, they have an electrical fan (14cm diameter and powered by a 12 volt battery), which produces airflow suction to capture mosquitoes approaching the traps [12, 13, 16]. The main difference between BGM and BGS is that BGM is hung upside down, 40cm above the ground, producing upward instead of downward airflow as produced by BGS (Fig 1). Both traps were used in the study.

### Recording equipment and software

The work was conducted at the Ifakara Health Institute’s Vector Laboratory (VectorSphere), located at Ifakara, Tanzania, using recording technology supplied by Noldus Information Technology, The Netherlands. The experiments were done inside a custom-built studio measuring 4m^2^ in area and lined with dark fiberglass netting on walls and floor to reduce reflection to the infrared cameras. Three high resolution Basler monochrome GigE cameras (Basler acA1300 - 60gmNIR) with complementary metal-oxide semiconductor (CMOS) sensors, coupled with three IR illuminators (Raytec RM25-120 Raymax 25 Infrared Illuminator—120–180° Beam, 26' Max IR Distance) with wavelength spectrum of 850 nm, were mounted on tripod stands (Velbon) inside the studio pointing at different angles to capture multiple images. The cameras were connected to a desktop computer in the control room, adjacent to the observation room, from where mosquito-responses could be observed. Mosquitoes were filmed using a Noldus Media Recorder (MR) 2.5, producing synchronous high-definition images from the cameras in MPEG-4 format.

**Fig 2:**
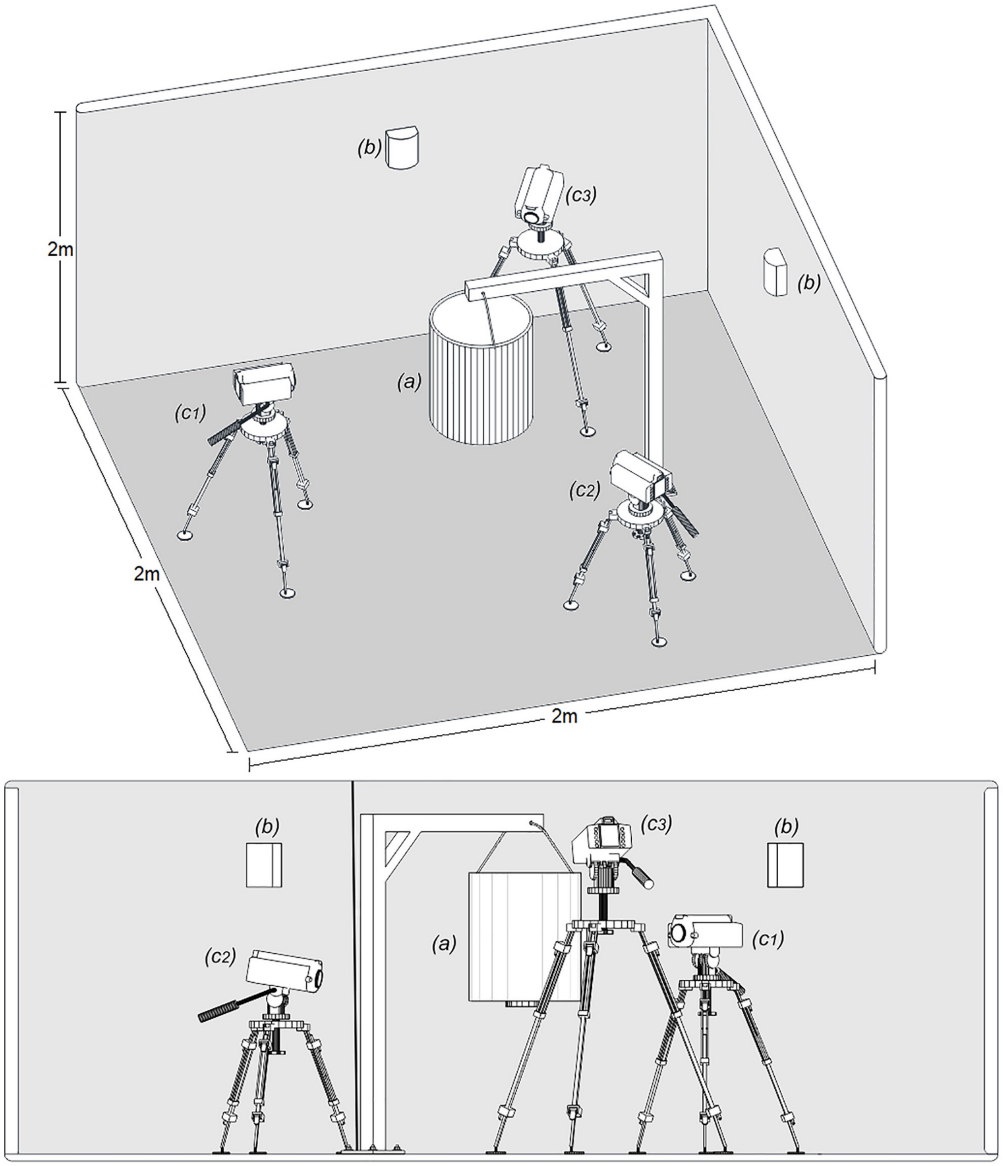
Experimental set-up in the video studio. The BG-Malaria trap (a) was installed in the center of the room, surrounded by Infrared illuminators (b) and three infrared video cameras (c1-3). The cameras were positioned to focus the lateral (c1), the entry point (c2) and the top (c3) of the trap. Observations on BG sentinel did not have top view.

The basic set-up of recording process is shown in Fig 2. The three infrared cameras were focused such that one camera produced the view of the white lid at the entrance of the traps (51cm x 36cm for BGS and 40cm x 36cm for BGM) (i.e., “entry point”), another viewed the lateral angles of the trap (36cm x 36cm) (i.e., “side”), and the third viewed the bottom of the trap (36cm diameter) (i.e., “top”) (Fig 1). The “top” view was applicable only for BGM, which was hung firmly upside down at the center of the studio using a wooden support and the bottom of the trap is viewed as the top. Together, entry point, side and top constituted the filming arena. The cameras captured up to 68 frames per second.

### Study procedures

The study consisted of three experiments, conducted nightly with 50 mosquitoes, each recording session lasting 30 minutes after a 10-minute acclimatization period inside the observation room. The experiments were replicated four times in different days with different batches of mosquitoes. At the end of every filming round, all mosquitoes were removed from the experimental room using a Prokopack aspirator [20].

#### Observations of flight behavior of host-seeking *An*. *arabiensis* mosquitoes towards the baited BG-Sentinel and BG-Malaria traps

Mosquito behaviour was recorded in relation to an individual trap positioned at the center of the studio. Traps were baited with the synthetic human odour, i.e., Ifakara blend [6], supplemented with industrial CO_2_ gas at a release rate of 500 ml/min. As the BGS lacked the top arena, the images for this experiment were only recorded for the entry point and sides of traps.

#### Assessing impact of installation height of BG-Malaria trap on flight behavior of host-seeking *An*. *arabiensis* mosquitoes

The BGM trap was positioned at the center of the studio, at heights of 20cm, 40cm and 80cm above the floor in different tests. In all tests, the trap was baited with the Ifakara blend plus CO_2_.

#### Comparing flight behaviors of host-seeking *An*. *arabiensis* around BG-Malaria baited with different attractants

The BGM was tested when baited with either the Ifakara blend plus CO_2_ or only CO_2_. In addition, an unbaited BGM was tested as control. The setups were evaluated separately and in different days to minimize the contamination between setups.

### Data analysis

The recorded footage was first processed using Ethovision XT 11.5 (Noldus Information Technology), to obtain heat maps and individual tracks of mosquitoes. The Ethovision software tracked up to 16 mosquitoes at a time per arena, but did not maintain individual identities of the mosquitoes throughout recording periods. The raw numeric data was then exported to R statistical software version 3.3.*2* [21], for further analysis using Generalized Linear Mixed Models (GLMMs). The function *glmer* was used to fit the GLMMs under the package *lme4* (Bates *et al*., 2015). Key parameters used to assess the responses of host-seeking mosquitoes towards BGS and BGM traps were: (1) velocity of the mosquito in the vicinity of the arena, (2) time spent in the vicinity of arena, (3) frequency of visits to the arena. Frequency of visits was modelled in Poisson distribution as a function of trap type as fixed effect, and replicate as random effect. On the other hand, time spent in the arena, and velocity of mosquitoes when visiting arena were modelled in Gamma distribution as a function of trap as fixed effect and replicate as random effect.

### Ethical considerations

This study was approved by both Ifakara Health Institute IRB (IHI/IRB/No: 34–2014) and the Medical Research Coordinating Council at the Tanzania National Institute of Medical Research (Certificate No. NIMR/HQ/R.8a/Vol.IX/1903).

## Results

### Heat maps and track visualization

**Fig 3:**
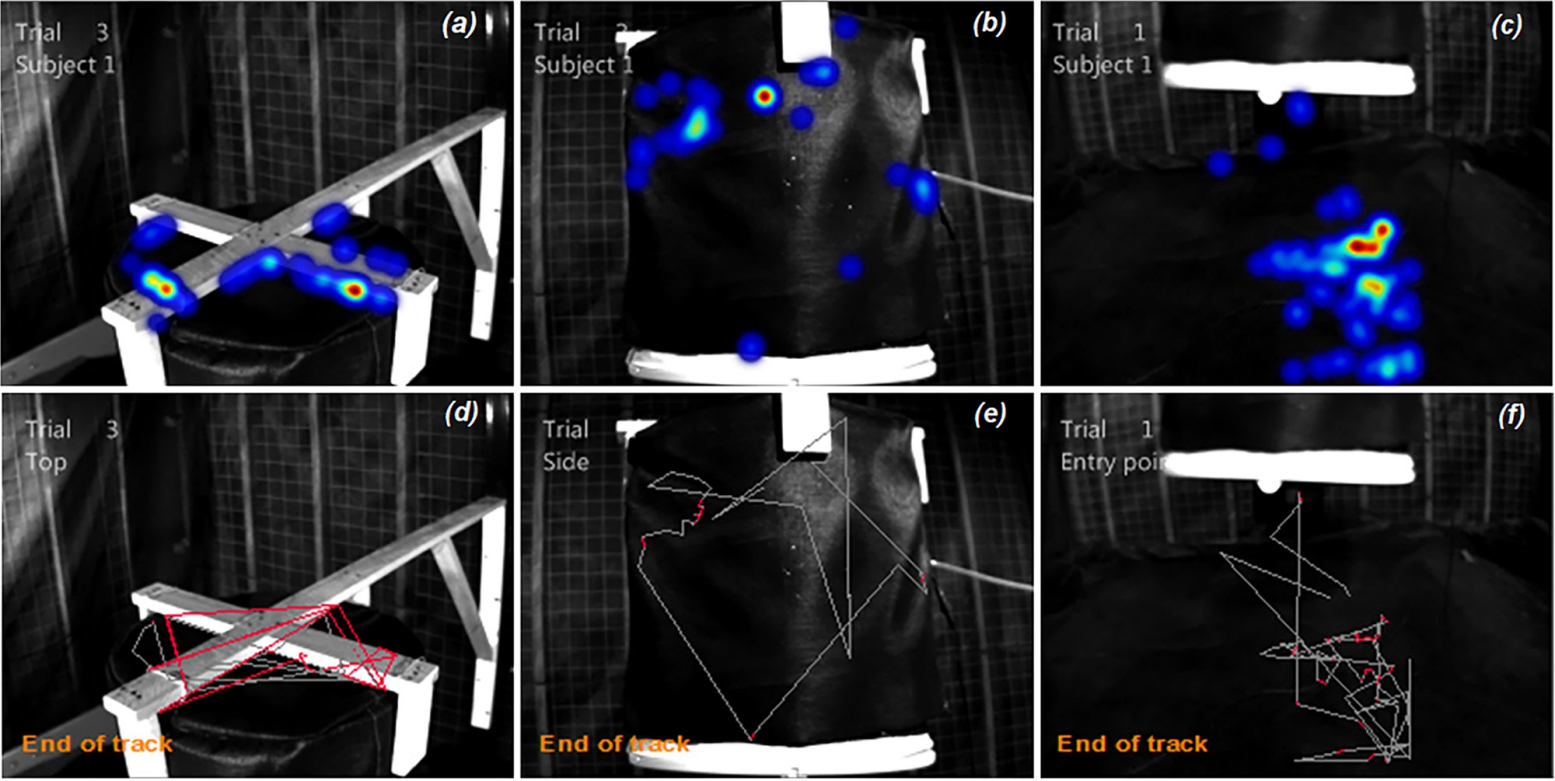
Heat maps and flight path of a single mosquito (Subject 1) around the top (*a* and *d*), lateral (*b* and *e*) and entrance (*c* and *f*) of the BG-Malaria trap baited with Ifakara blend plus CO_2_ set at 40cm height. Each heat map and flight path shown was created as a result of the mosquito activities on each arena during the experiment period (30 minutes). Different colors on the panels *(a)* to *(c)* represent frequency of visits in different places, from blue (least visited) to red (most visited). The red lines on the panels *(d)* to *(f)* means the patch taken by mosquitoes around the trap, while the grey lines shows the non-tracked path of mosquitoes reconstructed by the software.

Mosquito flight paths were constructed from the recorded footage, except for when the mosquitoes flew out of the arena or flight velocities were exceedingly high. An example of a heat map and tracks is shown in Fig 3, showing different colors for places visited least (blue) to those visited the most (red) (Fig 3*a*–3*c*). The tracked path was visualized in red color and the non-tracked segments of the entire path, as reconstructed using the software model, was visualized in grey color (Fig 3*d*–3*f*).

### Flight behavior of host-seeking *An*. *arabiensis* towards baited BG-Sentinel and BG-Malaria traps

Of the 400 mosquitoes released, 200 mosquitoes per trap separately, in the studio throughout the study period 45.5% (n = 91) and 53% (n = 106) of the mosquitoes visited the arenas (entry point and side) of the BGS and BGM traps, respectively. The frequency of mosquitoes visiting the entry point of the BGM was higher than in the BGS (p < 0.001). Comparing to the BGS, mosquitoes spent longer time on the entry point (p < 0.001) and side (p < 0.001) of the BGM. Moreover, flight velocity was higher when visiting both BGM entry point (p < 0.001) and BGM side (p < 0.001) compared to BGS (Table 1).

**Table 1:** Frequency of visits, flight velocity and time spent by host-seeking *Anopheles arabiensis* females around each arena of the BG-Malaria trap relative to the BG-Sentinel trap.

Arena	Variable	Trap	Median [IQR]	RR [95%: LC, UC]	P value
**Entry**	**Frequency (n)**	BG-Sentinel	15.00 [11.00, 21.00]	1	<0.001
		BG-Malaria	61.00 [27.00, 76.00]	3.34 [3.05, 3.66]	
	**Time (s)**	BG-Sentinel	0.20 [0.19, 0.21]	1	<0.001
		BG-Malaria	0.30 [0.28, 0.32]	2.19 [1.54, 3.11]	
	**Velocity (cm/s)**	BG-Sentinel	2.14 [0.93, 5.07]	1	<0.001
		BG-Malaria	13.73 [11.44, 15.27]	2.93 [1.96, 4.39]	
					
**Side**	**Frequency (n)**	BG-Sentinel	4.00 [3.00, 6.00]	1	<0.001
		BG-Malaria	7.00 [4.00, 12.00]	1.92 [1.62, 2.25]	
	**Time (s)**	BG-Sentinel	0.21 [0.19, 0.37]	1	<0.001
		BG-Malaria	0.31 [0.25, 0.50]	12.41 [6.37, 24.18]	
	**Velocity (cm/s)**	BG-Sentinel	0.80 [0.53, 1.73]	1	<0.001
		BG-Malaria	7.60 [2.35, 21.24]	10.86 [6.48, 18.19]	

Entry = arena showing the entry point of the traps;

Side = arena showing the lateral of the traps.

### Flight behaviors of host-seeking *An*. *arabiensis* towards BG-Malaria traps set at different heights

The BGM was tested at three different heights (20cm, 40cm and 80cm above the floor), to evaluate flight behaviors of mosquitoes around it. BGM traps set at 20cm height received more visits from the mosquitoes at the entry point than traps set at 40 cm (p < 0.001) and 80 cm (p < 0.001). However, no difference was found between traps installed at 40cm and 80cm (p = 0.670). More visits were observed on the side of the 20 cm BGM traps compared to the 40 cm (p < 0.05) and 80 cm traps (p < 0.001). For the top view, more visits were observed on traps set at 80cm height compared to those at 20cm (p < 0.001) and 40cm (p < 0.001), but there was no difference between traps installed at 40cm and 20cm (p = 0.133) (Table 2).

**Table 2:** Frequency of visits, flight velocity and time spent by host-seeking *Anopheles arabiensis* females around the arenas of the BG-Malaria trap installed at 40cm and 80cm heights relative to 20cm height.

Arena	Variable	Treatment	Median [IQR]	RR [95%: LC, UC]	P value
**Entry**	**Frequency (n)**	20 cm	161.0 [91.0, 237.0]	1	
		40 cm	61.0 [27.0, 76.0]	0.25 [0.24, 0.27]	<0.001
		80 cm	49.0 [26.0, 69.0]	0.23 [0.22, 0.24]	<0.001
	**Time (s)**	20 cm	0.32 [0.25, 0.34]	1	
		40 cm	0.31 [0.28, 0.32]	1.18 [0.83, 1.69]	0.347
		80 cm	0.09 [0.08, 0.10]	0.23 [0.16, 0.31]	<0.001
	**Velocity (cm/s)**	20 cm	1.52 [1.42, 2.53]	1	
		40 cm	13.73 [11.44, 15.27]	3.89 [2.68, 5.65]	<0.001
		80 cm	15.82 [8.54, 28.67]	7.66 [5.34, 11.00]	<0.001
					
**Side**	**Frequency (n)**	20 cm	13.00 [5.00, 17.75]	1	
		40 cm	7.00 [4.00, 12.00]	0.59 [0.52, 0.66]	<0.001
		80 cm	7.00 [4.00, 12.00]	0.56 [0.49, 0.64]	<0.001
	**Time (s)**	20 cm	0.21 [0.14, 3.61]	1	
		40 cm	0.32 [0.25, 0.52]	2.75 [1.34, 5.64]	<0.01
		80 cm	0.09 [0.08, 0.11]	0.03 [0.01, 0.06]	<0.001
	**Velocity (cm/s)**	20 cm	1.81 [1.42, 5.20]	1	
		40 cm	7.62 [2.35, 21.24]	3.52 [2.08, 5.95]	<0.001
		80 cm	1.67 [1.38, 2.54]	1.42 [0.81, 2.51]	0.220
					
**Top**	**Frequency (n)**	20 cm	12.00 [6.50, 15.25]	1	
		40 cm	8.00 [5.00, 12.00]	0.91 [0.81, 1.03]	0.133
		80 cm	45.00 [15.50, 70.50]	4.23 [3.87, 4.63]	<0.001
	**Time (s)**	20 cm	0.31 [0.23, 0.42]	1	
		40 cm	0.31 [0.26, 0.63]	7.35 [3.83, 14.08]	<0.001
		80 cm	0.08 [0.08, 0.09]	0.05 [0.02, 0.11]	<0.001
	**Velocity (cm/s)**	20 cm	20.72 [2.18, 68.57]	1	
		40 cm	7.62 [2.34, 21.24]	0.46 [0.28, 0.77]	<0.01
		80 cm	8.54 [2.53, 14.31]	0.30 [0.17, 0.54]	<0.001

Entry = arena showing the entry point of the trap;

Side = arena showing the lateral of the trap;

Top = arena showing the base of the trap.

The mosquitoes spent more time at the entry point when the BGM was installed at 20 cm. However, this was only significantly different from the BGM installed at 80 cm (p < 0.05) but not at 40cm. When the arena observed was the side of the BGM, traps installed at 40cm had longer visits than that those at 80cm (p < 0.001) and 20cm (p < 0.01). Similarly, the mosquitoes stayed longer at 20cm than at 80cm installations (p < 0.001). In the last arena observed, i.e., the top of the trap, mosquitoes also spent longer near traps installed at 40cm compared to 20cm (p < 0.001) and 80cm (p < 0.05) (Table 2).

Mosquitoes flew with significantly lower velocity when visiting the entry point of BGM traps at 20cm compared to those set at 40cm (p < 0.001) and 80cm (p < 0.001). Significant differences were also observed between traps at 40cm and 80cm (p < 0.05). In observations of the side arena, mosquitoes flew with lower velocity when visiting traps set at 20cm and 80cm, compared to traps set at 40cm (p < 0.001). Observing the top of the BGM, the velocity of the mosquitoes was higher when the trap was set at 20 centimeters, compared to 40 (p < 0.01) or 80cm (p < 0.001) (Table 2).

### Flight behavior of host-seeking *An*. *arabiensis* towards BG-Malaria trap baited with different attractants

The frequency with which mosquitoes flew near the entry points, on the sides and at the top of BGM trap was influenced significantly by the type of attractant used (Table 3). More mosquitoes visited the entry point when the BGM was baited with only CO_2_ (p < 0.001) or Ifakara blend plus CO_2_ (p < 0.001) than when the BGM was not baited. Significant differences were observed between the different baits (Ifakara blend plus CO_2_ or CO_2_ alone) (p < 0.001). When observing trap sides, fewer mosquitoes visited the unbaited BGM than BGM baited with Ifakara blend plus CO_2_ (p < 0.001). The BGM baited with the Ifakara blend plus CO_2_ also received more visits on the top, than the unbaited control (p < 0.001) and BGM baited with just CO_2_ alone (p < 0.001) (Table 3).

**Table 3:** Frequency of visits, flight velocity and time spent by host-seeking *Anopheles arabiensis* females around the arenas of the BG-Malaria trap baited with different lures relative to control (unbaited trap).

Arena	Variable	Treatment	Median [IQR]	RR [95%: LC, UC]	P value
**Entry**	**Frequency (n)**	Control	7.00 [4.00, 11.00]	1	
		CO_2_	65.0 [44.0, 125.0]	9.83 [8.70, 11.13]	<0.001
		IB + CO_2_	61.0 [27.0, 76.0]	6.71 [5.92, 7.60]	<0.001
	**Time (s)**	Control	0.42 [0.27, 2.07]	1	
		CO_2_	0.39 [0.35, 0.44]	0.03 [0.01, 0.05	<0.001
		IB + CO_2_	0.30 [0.28, 0.32]	0.05 [0.02, 0.10]	<0.001
	**Velocity (cm/s)**	Control	2.05 [0.70, 5.91]	1	
		CO_2_	12.75 [9.58, 15.24]	4.86 [2.89, 8.16]	<0.001
		IB + CO_2_	13.73 [11.44, 15.27]	2.64 [1.64, 4.24]	<0.001
					
**Side**	**Frequency (n)**	Control	3.00 [1.00, 7.00]	1	
		CO_2_	3.00 [2.00, 5.00]	0.96 [0.75, 1.24]	0.774
		IB + CO_2_	7.00 [4.00, 12.00]	2.02 [1.60, 2.54]	<0.001
	**Time (s)**	Control	1.18 [0.49, 2.84]	1	
		CO_2_	0.26 [0.21, 0.43]	0.59 [0.18, 1.94]	0.388
		IB + CO_2_	0.32 [0.25, 0.52]	0.06 [0.02, 0.17]	<0.001
	**Velocity (cm/s)**	Control	0.64 [0.21, 1.47]	1	
		CO_2_	5.97 [0.37, 24.68]	33.41 [15.43, 72.34	<0.001
		IB + CO_2_	7.62 [2.35, 21.24]	24.67 [11.72, 51.94]	<0.001
					
**Top**	**Frequency (n)**	Control	4.00 [2.00, 9.00]	1	
		CO_2_	8.00 [6.00, 12.00]	1.25 [1.06, 1.48]	<0.01
		IB + CO_2_	10.00 [7.75, 17.50]	2.14 [1.88, 2.44]	<0.001
	**Time (s)**	Control	0.43 [0.29, 12.64]	1	
		CO_2_	0.44 [0.28, 15.69]	0.49 [0.19, 1.29]	0.147
		IB + CO_2_	0.57 [0.35, 1.82]	0.49 [0.22, 1.08]	0.077
	**Velocity (cm/s)**	Control	2.76 [0.89, 16.96]	1	
		CO_2_	5.40 [0.77, 23.52]	0.87 [0.45, 1.67]	0.669
		IB + CO_2_	14.37 [2.74, 38.44]	1.75 [1.06, 2.90]	<0.05

Entry refers to the arena showing the entry point of the trap;

Side refers to the arena showing the lateral side of the trap;

Top refers to the arena showing the base of the trap;

CO_2_ = Trap baited with only CO_2_;

IB + CO_2_ = Trap baited with Ifakara blend plus CO_2_;

Control = unbaited trap.

Significant differences in the time spent on the entry point was observed, with less time spent in the CO_2_-baited BGM than in the unbaited BGM (p < 0.001) (Table 3). In comparison with the unbaited trap, mosquitoes spent less time on sides of BGM baited with Ifakara blend plus CO_2_ (p < 0.001). At the top of the trap however, no significant difference was found between all the treatments evaluated in time spent by mosquitoes (Table 3). Higher velocity was observed when mosquitoes visited the entry point of the BGM baited with only CO_2_ (p < 0.001) or Ifakara blend plus CO_2_ (p < 0.001) compared to unbaited control. Similar findings were observed in the side of the traps. Lastly, mosquitoes flew faster at the top of BGMs baited using Ifakara blend plus CO_2_, compared to the control (p < 0.05) (Table 3).

## Discussion

The need for exposure free methods to sample mosquitoes that can be as sensitive as human volunteer catchers is increasingly important. This is particularly urgent as countries seek to integrate effective surveillance programs as core-interventions in line with global policies [1]. Odour-baited traps are frequently used for vector surveillance and can constitute alternative tools for such surveillance on a large scale. The development of the BGM was through simple adaptation of commonly used BG-Sentinel trap (BGS), with the main difference being the upside down installation of the former, to provide inverted airflow orientation [13, 16]. When first tested, performance of BGM trap for sampling Brazilian malaria vectors in field tests was as good as the HLC [13]. To assess suitability in multiple sites, the BGM was then also evaluated in semi-field [16] and field conditions in Tanzania [15], to validate its performance as an efficient and safer method for collecting anophelines that can potentially replace the HLC. In both cases, the trap demonstrated good performance in terms of species diversities and physiological stages of the mosquitoes collected, even though the actual numbers did not match HLC, and the performance depended on the type of lure used [15]. This current study was designed to complement those earlier studies in Tanzania, and involved a series of experiments to assess how mosquitoes approach and enter the trap, and how long they stay in the vicinity of the trap when set at different heights or baited with different attractants. The videographic observations therefore also enabled demonstration of feasibility of such an approach for high-throughput evaluation and improvement of traps during development.

The findings of this video tracking largely match those observed in the field and semi-field tests. For example, Batista *et al*. [16] reported that twice as many *An*. *arabiensis* females were caught by the BGM than by the BGS in semi-field trials and the results presented here also show that more mosquitoes flew around the vicinities of the BGM than in the BGS. The frequency of mosquitoes flying, and the time they spent in the trap vicinity were also higher for BGM than in BGS. Different flight dynamics of mosquitoes observed around identical traps deployed in two opposite ways have been previously described by by Cribellier *et al*. [18], where these authors observed that mosquitoes flying downwards near the traps turned to fly upwards resulting in increased likelihood of capture by hanging traps, and higher likelihood of escape from standing traps. Such flight behaviour towards the hanging trap was consistent with our present findings, where mosquitoes approaching in downward flight then flew upwards around the BGM entry point.

A potentially important finding was that velocity in flight of mosquitoes around the BGM was higher than in the arenas of the BGS and that these velocities were highest at the entry point. When turning to fly upward, mosquitoes tend to accelerate their flight to perform upward-directed manoeuvres [18, 22]. On the other hand, as the trap entrance is the area with the highest air volume and speed due to the airflow produced by the trap fan, it is possible that this high suction airspeed directly influenced the flight velocity and the likelihood of mosquitoes being sucked into the trap. Since inverting the airflow in the BGM extends the area of high-speed flow more than 17 times [18], this may cause an increase in observed flight velocity.

This downward/upward flight dynamic was previously described in anthropophilic host-seeking *Anopheles* mosquitoes, which were guided by the host odour to land on the body parts that were closest to the ground [23]. After detecting an odour cue in a wind tunnel, *Aedes aegypti* mosquitoes spent most of the time flying near the ground to explore a visual object [24]. Our results showed a similar behaviour, as mosquitoes visited the BGM installed 20cm above the ground more than the traps positioned at greater heights. Mosquitoes also spent longer in the arenas of the lower set traps, i.e., with the entrance at 20cm and 40cm above the ground, and the time spent by mosquitoes in the entry point and side of the traps in both heights was not different from each other. Schmied et al. [25] also used the BGS to collect *Anopheles* and caught more mosquitoes than in an upward flow trap. Although the air current was downward, the BGS was installed below ground level, which may have affected its effectiveness as this original configuration in the present study received less visits by the mosquitoes compared with the BGM, which are an upside-down variant of the BGS. These findings indicate that mosquitoes most likely fly close to the ground, which in combination with the human odours present on the feet and convective air currents may account for the tendency of *Anopheles* to bite on the low on the host. Furthermore, the average velocity at which mosquitoes flew near the entry point of the BGMs decreased as the traps were set lower down. This result was consistent with the study of Cooperband & Carde [17], which reported that mosquitoes decelerated their flight when approaching the trap, adopting a sinuous flight, similar to the flight track demonstrated in the Fig 3*e* and 3*f*.

Through the combination of various host cues, such as odour, CO_2_ and visual features, odour-baited traps target the female mosquitoes in the host-seeking stage. The BGM has a black and white colour pattern and was originally conceived to use CO_2_ as bait [13]. As part of the BGM continuous optimization, other attractants like synthetic human odour were also evaluated in semi-field and field conditions, with the finding that CO_2_-baited traps caught similar numbers of *An*. *arabiensis* as traps baited with synthetic attractants [15, 16]. In this current study, more mosquitoes were attracted to the entry point of the CO_2_-baited BGM than the BGM baited with the synthetic attractant Ifakara blend plus CO_2_. The function of CO_2_ as an attractant for host-seeking mosquitoes has been amply described [26–28], and CO_2_ is considered an important flight activator when mosquitoes need to find a host [29–32]. Female mosquitoes can detect small changes in CO_2_ concentration in the air, using their sensory system [26, 33–35]. Dekker et al. [36] reported that in a wind-tunnel assay mosquitoes were induced to fly upwind by increasing the ambient concentration of CO_2_ by 0.5%. However, CO_2_ triggers a poor response in the attraction of highly anthropophilic mosquitoes [30, 37], which use a species-specific combination of human odours and CO_2_ [38–43]. *An*. *arabiensis* is an opportunistic species that feeds on both humans and animals [44, 45] and such opportunistic/zoophilic species respond well to breath [46, 47], which contains approximately 5% CO_2_. These previous findings about the role of CO_2_ in mosquitoes host-seeking behaviour are consistent with our results that displayed a greater attraction of *An*. *arabiensis* females to both baited BGMs over control (unbaited BGM).

Trap designs, height and attractants all influence mosquito activity in vicinity of the traps. Moreover, this activity can be readily visualized using infrared cameras to accelerate trap development and testing. One limitation of the present study was in the continuous tracking of mosquito flight paths, which was particularly challenging due to the high flight velocities in practice. Nonetheless, the software that we employed was able to reconstruct, and allow the visualization of, the non-tracked segments by mathematic modeling (e.g., Fig 3*c*–3*f*).

## Conclusion

Knowledge of vector flight behavior is of great importance to develop new tools that can successfully contribute to the fight against vector-borne disease. Since the conception of the BGM by adapting the BG-Sentinel trap, studies have been conducted to increase the trapping efficiency [14, 16]. In our study, the greater activity of host-seeking mosquitoes near BGM than BGS traps supports the proven superiority of BGM traps in field and semi-field settings. Moreover, the results we present here provide new insights into the capture mechanism of the BGM trap and will inform future improvement of this trap to the point where it can successfully replace the HLC.
